# *Janibacter melonis* bacteremia following autologous stem cell mobilization in lymphoma: a case report and literature review

**DOI:** 10.1186/s12879-025-12303-5

**Published:** 2025-12-12

**Authors:** Jinyan Liu, Ci Duan, Feng Li, Yan Man, Lin Tuo, Yilan Luo, Limei Li, Xun Lai, Youquan Zhou

**Affiliations:** 1 Department of Hematology, Yunnan Cancer Hospital, The Third Affiliated Hospital of Kunming Medical University, Peking University Cancer Hospital Yunnan, Kunming, China; 2 Department of Laboratory Medicine, Yunnan Cancer Hospital, The Third Affiliated Hospital of Kunming Medical University, Peking University Cancer Hospital Yunnan, Kunming, China

**Keywords:** Bacteremia, *Janibacter melonis*, Hematologic malignancy, Immunocompromised host

## Abstract

**Introduction:**

Infection is a major cause of morbidity and mortality in patients with hematologic malignancies. *Janibacter melonis* is a rare opportunistic pathogen capable of causing bacteremia even in healthy individuals, yet reports in hematologic malignancy are limited. This case represents one of the few documented infections in this context, with details on diagnostic confirmation, antimicrobial susceptibility, and treatment outcomes.

**Case presentation:**

A 37-year-old female with refractory diffuse large B-cell lymphoma developed *Janibacter melonis* bacteremia following autologous stem cell mobilization. The patient was immunocompromised as a result of prior therapy with a CD20 monoclonal antibody and intensive chemotherapy. She presented with fever and elevated inflammatory markers after consuming possibly spoiled kiwi fruit. Blood cultures identified *Janibacter melonis*, confirmed by 16 S ribosomal RNA gene sequencing. Antimicrobial susceptibility testing showed relatively weak activity of penicillins, cephalosporins, erythromycin, and clindamycin against the *Janibacter melonis* strain, whereas fluoroquinolones, aminoglycosides, tetracycline, glycopeptides, carbapenems, sulfonamides, rifampin, linezolid, and daptomycin had lower MICs; nitrofurantoin was inactive. The patient was treated successfully with cefoperazone–sulbactam, resulting in resolution of fever and normalization of inflammatory markers.

**Conclusions:**

This case highlights the importance of considering rare infections in immunocompromised patients, especially those with hematologic malignancies. Prompt microbiological and molecular diagnosis, combined with targeted therapy, is essential for good outcomes. This report also provides useful insights for managing *Janibacter melonis* infections.

## Introduction

The genus *Janibacter* belongs to the class *Actinobacteria*, order *Actinomycetales*, and family *Intrasporangiaceae*. It is a Gram-positive, aerobic bacterium with variable oxidase and positive catalase activity, exhibiting a characteristic rod-coccus cycle and resembling the two-faced Roman god Janus during its growth [1]. *Janibacter* is considered as an opportunistic pathogen. It is found in various environmental conditions, including polluted soil, river water, non-saline alkaline groundwater, and air contaminated by wastewater treatment plants or industrial activities, as well as in spoiled fruits and infected insects [2–4]. Species of the genus *Janibacter*, including *J. terrae*, *J. hoylei*, *J. indicus*, *J. massiliensis sp. nov.*, and *J. melonis*, have been isolated from humans, confirming the occurrence of *Janibacter* infection in human patients. Case reports indicate that *Janibacter* species, including *J. terrae*, *J. hoylei*, and *J. indicus*, can cause bacteremia, particularly in immunocompromised individuals such as those with acute leukemia, infants, and the elderly. A fatal case of *J. terrae* bacteremia even occured in a patient with severe immunosuppression [1, 5–7]. Additionally, *Janibacter* species have also been isolated from clinical specimens in cases of vaginitis, psoas abscess, and resected aortic valve from patients with aortic valve stenosis [8–10].

*Janibacter melonis* (*J. melonis*), a significant species of the genus *Janibacter*, is recognized as a rare human pathogen. Despite limited research, available studies indicate its pathogenic potential. A case has been reported of *J. melonis* causing bacteremia in a healthy middle-aged individual following skin trauma [11]. Furthermore, the isolation of *J. melonis* from the duodenal mucosa of patients with celiac disease suggests its potential role as an intestinal pathogen [12]. Patients with hematological malignancies often experience immune deficiencies due to disease pathology and antitumor therapies, leading to a higher risk of infection [13]. However, no cases of *J. melonis* infection have been reported in the patients with hematological malignancies, and there is currently no clinical guidance available for the diagnosis and treatment of such infection in this patient population.

Here, we report a case of a patient with refractory diffuse large B-cell lymphoma (DLBCL) who developed a fever following autologous hematopoietic stem cell mobilization and collection. *J. melonis* was isolated and identified from the patient’s peripheral blood culture, and the bacteremia was successfully managed with antimicrobial therapy. This case aims to provide clinical insights for the diagnosis and treatment of similar infection of *J. melonis* in the future.

## Case presentation

A 37-year-old female was diagnosed with double-expressor diffuse large B-cell lymphoma(non-germinal center subtype, stage IV), with an International Prognostic Index (IPI) score of 4, indicating high risk. After chemotherapy with two cycles of first-line immunochemotherapy, the patient did not achieve a partial response, she was diagnosed with refractory DLBCL. She then received five cycles of second-line intensive immunochemotherapy with Bruton’s tyrosine kinase inhibitor (Zanubrutinib), achieving complete remission (CR). Since she is a young patient with high-risk refractory DLBCL, we have planned consolidation therapy with autologous stem cell transplantation for her. She was hospitalized in the Department of Hematology, Yunnan Cancer Hospital, and received a stem cell mobilization regimen with rituximab, etoposide, and cytarabine (R-EA) plus G-CSF from February 26 to March 19, 2024. During this period, she developed *Escherichia coli* sepsis and COVID-19 infection, along with grade IV bone marrow suppression and neutropenia. After treatment with cefoperazone-sulbactam and Paxlovid, her manifestations of infection improved. Stem cells were successfully collected on March 19, 2024, and she was discharged on March 20, 2024 after recovery from neutropenia. Two days after discharge, the patient developed a fever with temperature of 38 °C with chills but no other symptoms, she presented to our hospital the next day with persistent low-grade fever. As shown in Table 1, initial blood tests revealed an elvated white blood cell count (37.13 × 10^9/L) with higher band neutrophils (14%) and lower lymphocytes (4%). Lymphocyte subset analysis revealed a marked lymphopenia, along with a significant reduction in B cells. Serological tests indicated a significant elevation in inflammatory markers, including procalcitonin, C-reactive protein, interleukin-6, and interleukin-10.

**Table 1:** Laboratory test characteristics

Laboratory values	Measured	Normal range
WBC (x10^9^/L)	37.13	3.5–9.5
Hb (g/L)	80	115–150
PLT (x10^9^/L)	102	125–350
Band neutrophils (%)	14	1–5
Segmented neutrophils (%)	48	40–75
Promyelocyte (%)	3	-
Myelocyte (%)	12	-
Metamyelocyte (%)	9	-
Monocytes (%)	9	3–10
Lymphocytes (%)	4	20–50
B lymphocytes (%)	0.1	5–18
B lymphocytes counts (cell/ul)	1	90–560
T lymphocytes (%)	80.2	61.5–76.3
T lymphocytes counts (cell/ul)	584	955–2860
T-helper/T-suppressor cell ratio	1.18	1.19–2.27
Procalcitonin (ng/ml)	7.46	≤ 0.05
C-reactive protein	43.31	≤ 6.0
Interleukin-6 (pg/ml)	44.42	≤ 5.4
Interleukin-10 (pg/ml)	36.86	≤ 12.9

**Fig. 1:**
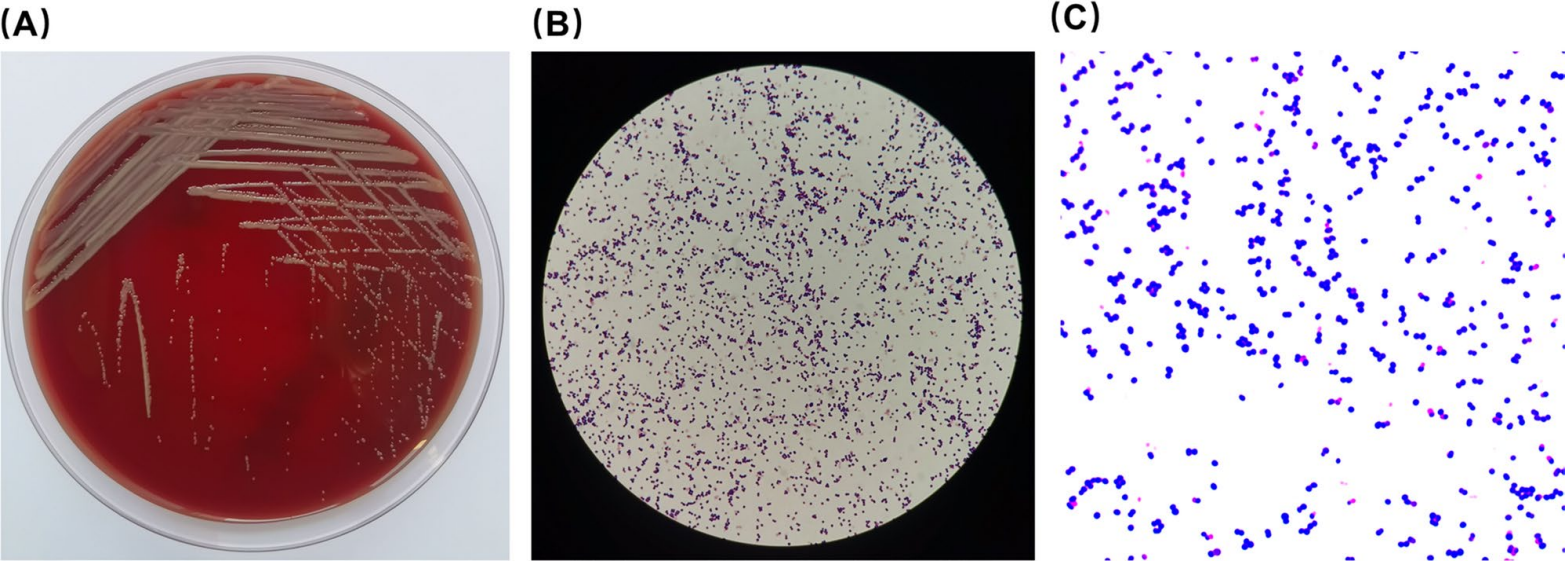
Morphology of *Janibacter melonis* isolated from the patient’s blood culture. (**A**) Blood agar plate after 48 h of aerobic incubation at 37 °C with 5% CO2. (**B**) Initial 1000× oil immersion Gram stain image captured through the eyepiece with a mobile phone. (**C**) 1000× oil immersion Gram stain image acquired by the microscope camera

**Fig. 2:**
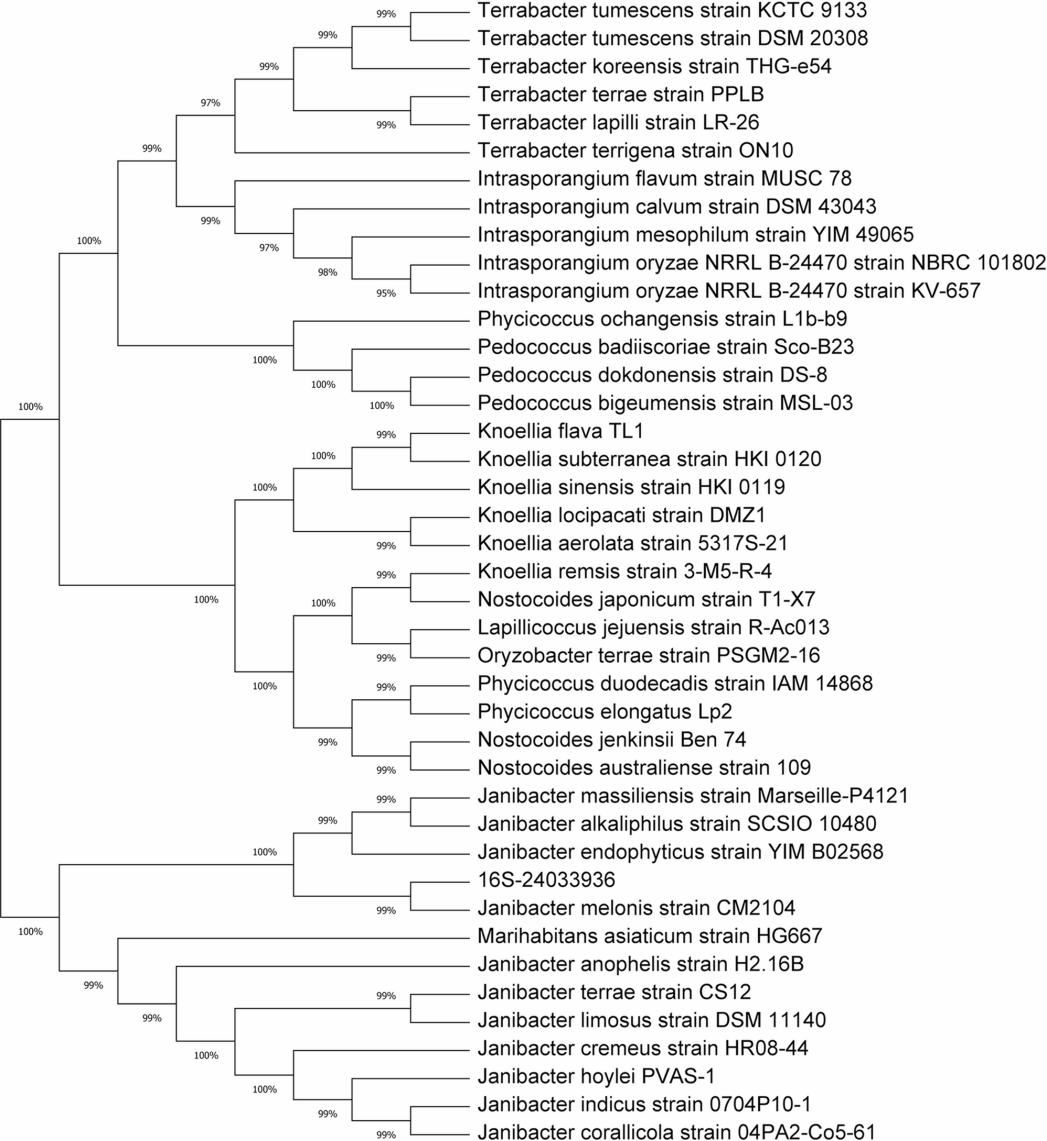
The phylogenetic tree based on the 16 S rRNA gene showed that the strain shares 99% phylogenetic similarity with *Janibacter melonis*

Peripheral blood cultures were obtained from the patient the day after fever onset. The blood culture aerobic bottle was positive after being cultured for 86.8 h. The culture is transferred to a blood agar plate for further cultivation. After 48 h of aerobic incubation at 37 °C with 5% CO_2_, colonies that were smooth, round, convex, and creamy in color were observed (Fig. 1A). Gram staining of the colonies identified them as gram-positive cocci (Fig. 1B and C). Phylogenetic analysis was performed using MEGA software by comparing the 16 S rRNA sequence of the laboratory-obtained strain with those of closely related strains retrieved from NCBI. The Neighbor-Joining method was used to construct the phylogenetic tree. The results showed that the laboratory-obtained strain belongs to the genus Janibacter and clusters within the same branch as *Janibacter melonis* strain CM2104, with a phylogenetic similarity of 99%, the colonies were identified as *Janibacter melonis* (*J. melonis*) (Fig. 2).

**Fig. 3:**
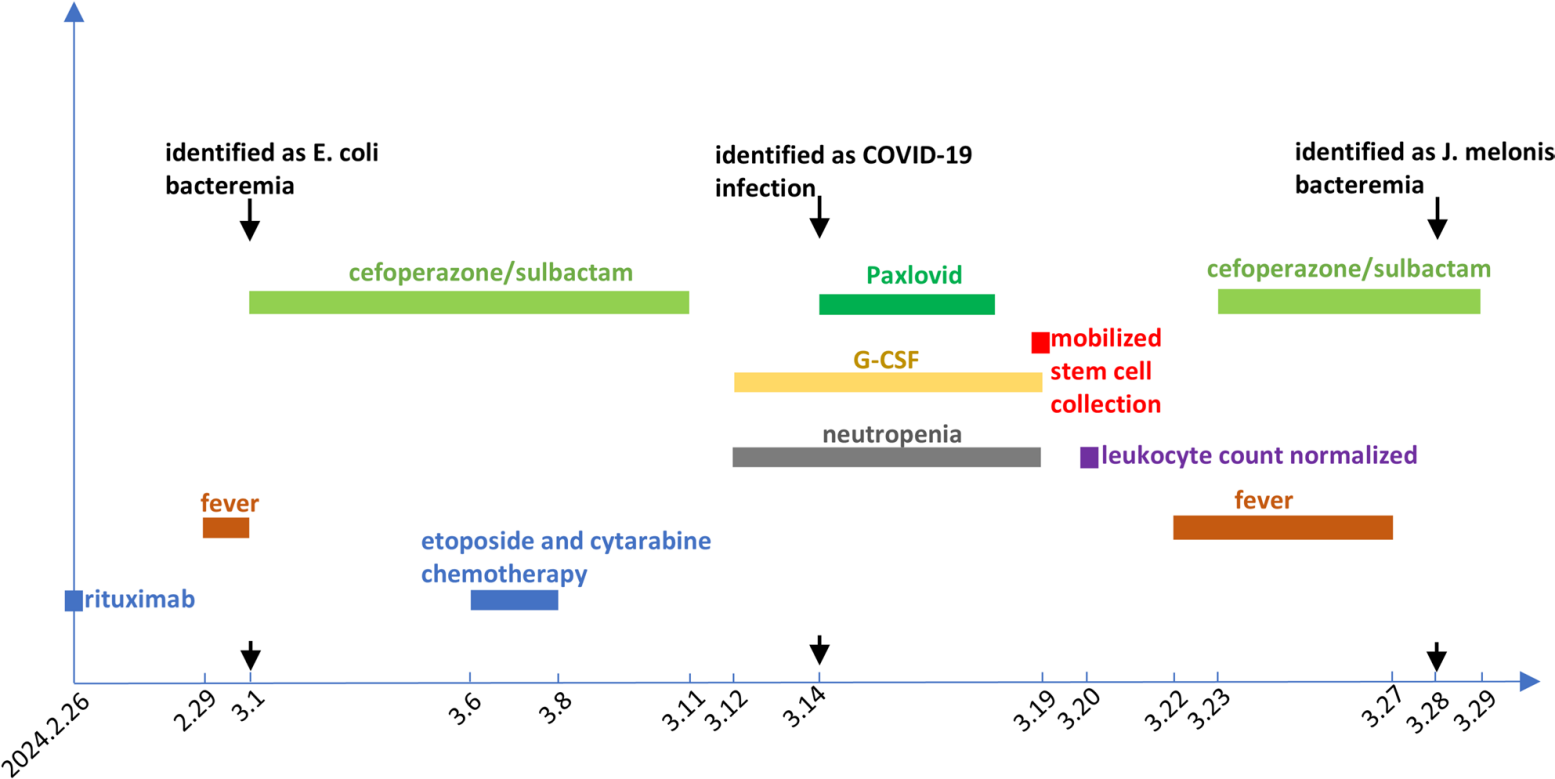
Timeline of the clinical course of the patient. Rituximab was administered on February 26, 2024. Fever developed on February 29 and March 1, with blood cultures positive for Escherichia coli; cefoperazone–sulbactam was given from March 1 to March 11. EA chemotherapy was administered from March 6 to March 8. Neutropenia developed on March 12, and G-CSF was administered for 8 days to support leukocyte recovery and stem cell mobilization. COVID-19 infection was confirmed on March 14 and treated with oral Paxlovid for 5 days. Peripheral blood stem cells were successfully collected on March 19, and the patient was discharged on March 20 with recovery from neutropenia. Two days later, she developed recurrent fever and was readmitted on March 23; empirical cefoperazone–sulbactam therapy was initiated for 7 days. She became afebrile after 5 days, and blood cultures were confirmed positive for *Janibacter melonis* on March 28

We performed broth microdilution testing using 24 commonly used antibiotics active against Gram-positive cocci and report only the MIC values (Table 2). Human infections caused by *Janibacter melonis* are extremely rare, and no antimicrobial susceptibility testing interpretive criteria are currently provided by either CLSI or EUCAST for this organism. Because no interpretive criteria exist and clinical validation is lacking, these agents cannot be categorized as susceptible, intermediate, or resistant. The MIC results are provided solely to inform potential empirical treatment decisions. Based on the MIC ranges alone, penicillins, cephalosporins, erythromycin, and clindamycin showed relatively weak in vitro activity, while fluoroquinolones, aminoglycosides, tetracycline, glycopeptides, carbapenems, sulfonamides, rifampin, linezolid, and daptomycin demonstrated lower MICs. Nitrofurantoin showed no activity. In fact, before the antibiotic susceptibility results were available, the patient was treated with cefoperazone/sulbactam (2:1 ratio) at 3 g every 12 h (total daily dose, 6 g) for 5 days. Subsequently, her fever resolved, and both procalcitonin and C-reactive protein levels returned to normal. Interestingly, upon further inquiry into the patient’s history of pathogen exposure, it was revealed that the patient had a history of exposure to a suspiciously spoiled kiwi fruit the day before the fever onset. The family members who shared the kiwi fruit experienced diarrhea without fever, while the patient presented only with fever and no gastrointestinal symptoms. A schematic timeline illustrating the key clinical events, treatments, and microbiological findings is shown in Fig. 3.

**Table 2:** Antimicrobial susceptibility testing results of *Janibacter Melonis* (MIC values)

Antimicrobial agent	MIC (µg/mL)
Penicillin	4
Ampicillin	8
Amoxicillin/clavulanate	8/4
Cefuroxime	4
Ceftriaxone	8
Ceftazidime	32
Cefoperazone	8
Cefoperazone/sulbactam	8/4
Cefepime	16
Meropenem	≤ 0.25
Rifampin	≤ 0.5
Linezolid	≤ 2
Daptomycin	≤ 1
Ciprofloxacin	2
Levofloxacin	≤ 2
Moxifloxacin	≤ 0.5
Nitrofurantoin	≥ 128
Erythromycin	4
Clindamycin	4
Vancomycin	≤ 0.5
Teicoplanin	≤ 2
Tetracycline	1
Trimethoprim–sulfamethoxazole	≤ 0.5/9.5
Gentamicin	≤ 2

Notes: MIC values were determined using the broth microdilution method. No CLSI or EUCAST breakpoints exist for *Janibacter melonis*, susceptibility categories (susceptible, intermediate, resistant) are therefore not assigned

## Discussion

Patients with hematologic malignancies often have immunodeficiency and a high risk of infection, with severe infection being a major cause of mortality. The genus *Janibacter* is a rare opportunistic pathogen that can cause bacteremia, particularly in immunocompromised individuals, and may be fatal in severe cases. However, there is a lack of clinical data and management experience regarding *Janibacter melonis* species infection in patients with hematologic malignancies. This report presents a case of *Janibacter melonis* bacteremia in a patient with refractory diffuse large B-cell lymphoma following autologous hematopoietic stem cell mobilization.

The genus *Janibacter* belongs to the order *Actinomycetales* and was first described in 1997 by Martin and his colleagues, who isolated and identified the bacteria from sludge samples in a wastewater treatment plant. These organisms were characterized as aerobic, Gram-positive, irregularly shaped rods [14]. Among them, *Janibacter melonis*(*J. melonis*) is an important species, was classified as an opportunistic pathogen, and first isolated from a spoiled oriental melon. It showed that *J. melonis* was observed microscopically as aerobic, Gram-positive, non-acid-fast, non-motile, non-spore-forming cocci [3], while another report described isolated *J. melonis* as Gram-negative coccobacilli [11]. In our case, *J. melonis* was isolated from the patient’s peripheral blood culture after approximately two days of aerobic incubation at 37 °C on non-selective agar, forming 1–3 mm, round, smooth, raised, glossy, cream-colored colonies. Microscopic examination revealed it as a Gram-positive cocci. Besides, based on the 16 S rRNA gene, the phylogenetic tree showed that the strain shares 99% phylogenetic similarity with *Janibacter melonis*, indicating a high genetic relatedness between the laboratory-obtained strain and *Janibacter melonis*.

Infection is a common and life-threatening complication in patients undergoing chemotherapy and hematopoietic stem cell transplantation (HSCT) for hematologic malignancies. The incidence of infection varies, reaching over 80% in acute leukemia, around 20–50% in myelodysplastic syndrome (MDS), neutropenic fever occured in 10–20% of patients treated for lymphoma, multiple myeloma (MM) patients had a 5-fold risk of developing a clinically significant infection compared to matched controls [15–18]. Studies reported that up to 90% of patients experienced infection following high-dose chemotherapy (HDC) and autologous stem cell transplantation (ASCT) [19]. Compared to patients with solid tumors, those with hematologic malignancies have a higher risk of infection due to more severe immune suppression [20–22]. This results from malignant immune cells, bone marrow infiltration, and impaired hematopoiesis [23–25]. High-dose chemotherapy, stem cell transplantation, immunotherapy, and targeted therapies further weaken both innate and adaptive immune responses by severely suppressing bone marrow function, which leads to pancytopenia, damaging skin and mucosal barriers, disrupting microbial balance [24, 26–28]. In addition, prolonged hospital stays and frequent invasive procedures further increase the risk of hospital-acquired infections in these patients [29]. In this case, the patient had a history of multiple infections prior to the *Janibacter* bacteremia, including *Escherichia coli* bacteremia and COVID-19, along with neutropenia during the mobilization of hematopoietic stem cells with the rituximab-EA immunochemotherapy. Although the patient was not neutropenic during the *J. melonis* bacteremia, the use of CD20 monoclonal antibodies and chemotherapy can lead to B-cell depletion, damage to mucosal barriers, and microbial dysbiosis, thereby compromising both cellular and humoral immunity. During the *J. melonis* bacteremia, we observed a marked reduction in both T and B lymphocytes, with B cells comprising only 0.1% of the total lymphocyte population. These findings suggest a potential link between the infection of *Janibacter melonis* and impaired immune function.

In fact, *J. melonis* is classified as opportunistic pathogen, previous reports on *J. melonis* infection are limited. Case reports indicated that *J. melonis* may cause bacteremia through skin injuries or act as a gut pathogen, being present in the duodenal mucosa of a celiac disease patient [11, 12]. In our case, this patient with DLBCL who developed *J. melonis* bacteremia had a history of consuming possibly spoiled kiwi fruit. Although the patient did not develop diarrhea like their family members who shared the same kiwi fruit, she presented with low-grade fever and elevated inflammatory markers. *J. melonis* was isolated from her blood after near three days of culture. Given the patient’s immunocompromised state and exposure history, we believe this episode represents a true infection and likely originating from the spoiled fruit. Reports of *J. melonis* infection are extremely limited, and its incubation period remains poorly defined. In this case, the patient consumed the kiwi fruit approximately 24 h before the onset of chills and low-grade fever, whereas family members who ingested the same fruit experienced only self-limited diarrhea within hours. Although the exact source of infection cannot be definitively confirmed, this timeline suggests that the incubation period for *J. melonis* bacteremia may be relatively short, particularly in immunocompromised individuals. Additional cases are needed to better characterize the incubation period and pathogenic potential of this rare organism.

This patient’s fever was resolved after five-day empirical treatment of cefoperazone-sulbactam, with a corresponding reduction in inflammatory markers. Antibiotic susceptibility testing showed that penicillins, cephalosporins, erythromycin, and clindamycin exhibited relatively weak in vitro activity against the *J. melonis* strain, whereas fluoroquinolones, aminoglycosides, tetracycline, glycopeptides, carbapenems, sulfonamides, rifampin, linezolid, and daptomycin demonstrated lower MIC values. Nitrofurantoin showed no detectable activity. Among the cephalosporins tested, cefoperazone and cefoperazone–sulbactam exhibited relatively weak antibacterial activity. Due to the strain’s slow growth, the antimicrobial susceptibility results took longer to obtain, and since the treatment with cefoperazone-sulbactam is effective, the treatment regimen was not adjusted according to the susceptibility results. Although ceftriaxone-sulbactam exhibited relatively weak antimicrobial activity against *J. melonis* in vitro, it demonstrated effective anti-infective efficacy in the patient. This observation suggests that infections caused by low-virulence organisms may respond clinically to antibiotics even when laboratory activity appears limited. Moreover, no established susceptibility breakpoints exist for rare organisms such as *J. melonis*, making interpretation of in vitro results challenging. Clinicians should therefore consider the overall clinical context, host immune status, and pharmacokinetic–pharmacodynamic properties when selecting therapy for rare pathogens.

## Conclusion

In conclusion, infection is the most common complication in treating hematologic malignancies, increasing treatment complexity, mortality, and healthcare costs. It is critical to address infection risks throughout the entire course of high-dose chemotherapy and autologous stem cell transplantation. In addition to common bacterial, fungal, and viral infections, awareness of rare pathogens should also be heightened. In this case, we report a patient with refractory diffuse large B-cell lymphoma who developed *Janibacter melonis* infection after stem cell mobilization, likely caused by ingesting spoiled fruit. Fever and bacteremia were the primary clinical manifestation, the pathogen *Janibacter melonis* was isolated via blood cultures and identified by gene sequencing, and the antimicrobial susceptibility data for *Janibacter melonis* were aslo provided. Overall, this case provides valuable diagnostic and therapeutic experience for managing such rare pathogen infection in immunocompromised patients, including those with hematologic malignancies.

## Data Availability

The data supporting this study’s findings are available from the corresponding author upon reasonable request.
